# Use of Mixed Reality in Neuro-Oncology: A Single Centre Experience

**DOI:** 10.3390/life13020398

**Published:** 2023-01-31

**Authors:** Swati Jain, Yujia Gao, Tseng Tsai Yeo, Kee Yuan Ngiam

**Affiliations:** 1 Division of Neurosurgery, University Surgical Cluster, National University Health System (NUHS), 1E, Kent Ridge Road, Singapore 119228, Singapore; 2 Division of Hepatobiliary & Pancreatic Surgery, University Surgical Cluster, National University Health System (NUHS), 1E, Kent Ridge Road, Singapore 119228, Singapore; 3 Division of Endocrine & Thyroid Surgery, University Surgical Cluster, National University Health System (NUHS), 1E, Kent Ridge Road, Singapore 119228, Singapore

**Keywords:** mixed reality, HoloLens 2, neuronavigation

## Abstract

(1) Background: Intra-operative neuronavigation is currently an essential component to most neurosurgical operations. Recent progress in mixed reality (MR) technology has attempted to overcome the disadvantages of the neuronavigation systems. We present our experience using the HoloLens 2 in neuro-oncology for both intra- and extra-axial tumours. (2) Results: We describe our experience with three patients who underwent tumour resection. We evaluated surgeon experience, accuracy of superimposed 3D image in tumour localisation with standard neuronavigation both pre- and intra-operatively. Surgeon training and usage for HoloLens 2 was short and easy. The process of image overlay was relatively straightforward for the three cases. Registration in prone position with a conventional neuronavigation system is often difficult, which was easily overcome during use of HoloLens 2. (3) Conclusion: Although certain limitations were identified, the authors feel that this system is a feasible alternative device for intra-operative visualization of neurosurgical pathology. Further studies are being planned to assess its accuracy and suitability across various surgical disciplines.

## 1. Introduction

Mixed reality (MR) technology has opened new avenues for planning, visualization, and education in surgery [1,2,3,4]. We have made a significant leap forward from initial virtual reality (VR) systems. Zhang et al. [5] has very eloquently described the differences between virtual reality, augmented reality, and mixed reality. VR is a pure virtual digital picture, whereas AR is a virtual digital picture plus naked eye reality, and MR is a virtual digital picture plus digital reality. The difference between MR and AR is that MR allows you to see the reality that is invisible to the naked eye through a camera. Augmented reality only superimposes the virtual environment regardless of reality itself. VR has provided a new display technology but was criticized for its multiple limitations. This was followed by augmented reality (AR), which provided some hope of incorporating new visualization systems in neurosurgery for training and education. Kockro et al. [6,7] developed an AR-based technology called DEX-Ray which provided an accurate and real-time video-based augmented reality display. The system could be seamlessly integrated into the surgical workflow. The see-through effect revealing 3D information below the surgically exposed surface proved to be of significant value, especially during the macroscopic phase of an operation, providing easily understandable structural navigational information. Navigation in deep and narrow surgical corridors was limited by the camera resolution and light sensitivity. However, a lack of interest from the neurosurgical community and availability of neuronavigation systems prevented this state-of-the-art technology from becoming a norm in the neurosurgical operating theatres.

Neurosurgical operations require significant information about spatial comprehension [8,9,10]. Due to the proximity of tumours to critical vascular and neural structures, it is important to ensure that the tumour is well localized. Some of the tumours are very deep seated, which requires adequate localization to prevent damage to the normal brain during the approach to the tumour. Intra-operative neuronavigation is currently an essential component to most neurosurgical operations [11,12]. Pre-operative surgical planning, intra-operative localization of deep-seated tumours, stereotactic biopsies and determining intra-operative resection margins for intrinsic tumours are some of the common applications of intra-operative navigation. Advancements in imaging have furthered its usage, such as planning minimally invasive surgeries and mapping white matter tracts [10]. 

However, there are several disadvantages that are associated with currently available systems [8,13,14,15,16]. Firstly, there is only the 2D projection of images on the screen requiring the surgeon to translate into a mental 3D image during planning. While some systems have incorporated the 3D reconstruction, it is rigid based on the pre-op planning of surgery. These systems only allow for visualization in the axial, sagittal and coronal planes. There are no further degrees of freedom, often limiting the visualization of the target tumour. Secondly, any errors in navigation after preparation and draping require re-registration and re-starting of the entire process. Errors of a few millimetres may result in missing the target lesions, especially in stereotactic biopsies, or may lead to injury of critical neurovascular structures. Lastly, intra-operative instruments or their paths of insertion cannot be visualized unless they have been pre-registered with the navigation system used. 

Recent progress in mixed reality (MR) technology has attempted to overcome the disadvantages of the neuronavigation systems, allowing the surgeon to superimpose a 3D-rendered image onto the patient’s anatomy [10,17,18,19]. Its utility has been limited to education and occasional intra-operative usage [20]. Several studies have recently shown its potential in aiding as an adjunct to neuronavigation. 

We present the first experience using the HoloLens 2 (Microsoft Corporation, Redmond, WA, USA) in neuro-oncology surgery. HoloLens 2 is the second generation of mixed-reality devices developed by Microsoft and represents a huge technological leap from first-generation devices. Three-dimensional renderings are performed using Virtual Surgery Intelligence (apoQlar GmbH, Hamburg, Germany) software, allowing the clinician to have full interaction capabilities with the images. Using the combination of HoloLens 2 and VSI, we evaluate the utility and feasibility of MR image rendering as a comparison to the current available neuronavigation systems. We have evaluated the feasibility of using the MR device across different locations of tumours (superficial vs. deep) and the possibility of using newer imaging adjuncts such as tractography along with conventional imaging. 

## 2. Materials and Methods

Three patients were recruited for the purpose of this case series. No identifiable patient data were used for this study. All patients were diagnosed with an intracranial tumour that required surgical resection. They underwent MRI imaging using standard pre-defined protocol for neuronavigation. These were 1 mm fine-cut imaging which are used as standard of care for intra-operative neuronavigation in our institution. No changes in the imaging modalities were performed for the purposes of the study. These images were transferred to a CD rom. 

The imaging files were uploaded onto the Microsoft Azure Cloud. The connectivity to the Microsoft Azure Cloud allowed for complex cloud processing along with the capabilities of the in-built processor and the ability for embedding artificial intelligence (AI) and machine learning algorithms that run in the background. The images were anonymized prior to uploading and processing. For the purposes of this study, we used Virtual Surgery Intelligence (VSI) software. Virtual Surgery Intelligence (VSI) provided the software capability for image rendering and real-time overlay during operation. It converted the MRI files in Digital Imaging and Communications in Medicine (DICOM) standard into a 3D rendering. VSI did not require modification of imaging protocols for rendering of the images. Hence, this software was used for the purposes of the study to look at the feasibility of using mixed reality in neurosurgery theatres. 

All patients underwent the same procedure to evaluate the feasibility of using HoloLens 2. All patients were placed on a Mayfield clamp in the desired position. Using the optical navigation module of Stealth S8, patients were registered using the pre-operative MRI images. Surface landmarks were checked to ensure that registration was accurate and acceptable for the purposes of the surgery. Subsequently, the primary user of the HoloLens 2 donned the device. The 3D-rendered image was then moved manually to overlay with the patient’s head. This was achieved using surface landmarks such as the pinna, nasion and tip of the nose. The limitations of manual overlay are discussed later. VSI software retained the spatial memory of the image overlaid on the patient’s skull. The surgical incision and craniotomy were decided based on neuronavigation. 

The surgical team proceeded to cleanse and drape the patient as part of the standard surgical preparation. No additional exposure was performed for the purposes of assessing the feasibility of the device and MR. Neuronavigation accuracy was rechecked with standard extracranial landmarks (medial and lateral canthi, nasion, external auditory meatus). Subsequently, the primary user of HoloLens 2 rechecked the overlaid 3D-rendered image. As the purposes of the study were only to assess the feasibility of usage of such a device intra-operatively, no further parameters were recorded. We have assessed our accuracy of MR in comparison to standard neuronavigation systems in another publication of our group (awaiting publication, has been accepted in Neurosurgery Open, 2023, https://www.repository.cam.ac.uk/handle/1810/345362, accessed on 27 January 2023). 

## 3. Results

### 3.1. Case 1

**Figure 1:**
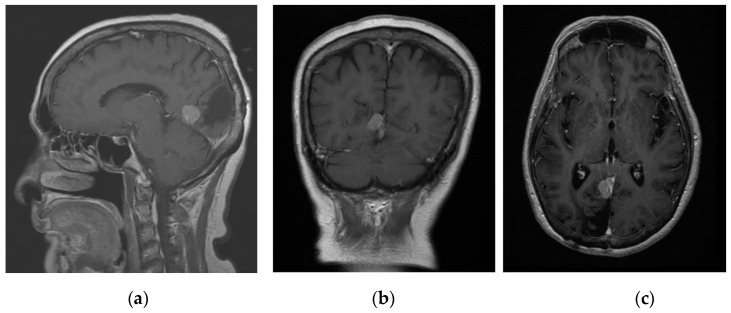
Contrasted T1 MRI: (**a**) sagittal, (**b**) coronal, and (**c**) axial planes showing recurrence of the lesion at the surgical bed, 1.5 cm in maximum diameter.

The first patient was a 72-year-old female with known history of right occipital parafalcine meningioma. The histology was consistent with that of atypical meningioma, WHO grade II. During follow up, she was noted to have recurrence of the lesion at the surgical bed, 1.5 cm in maximum diameter (Figure 1). The patient agreed for surgical resection of the lesion. As shown in Figure 1, patient’s tumour was deep seated, and an accurate neuronavigation was required to ensure that the tumour was resected. Standard neuronavigation MRI images were obtained for intra-operative planning. These images were then uploaded onto the HoloLens 2 system for rendering into a 3D image. 

**Figure 2:**
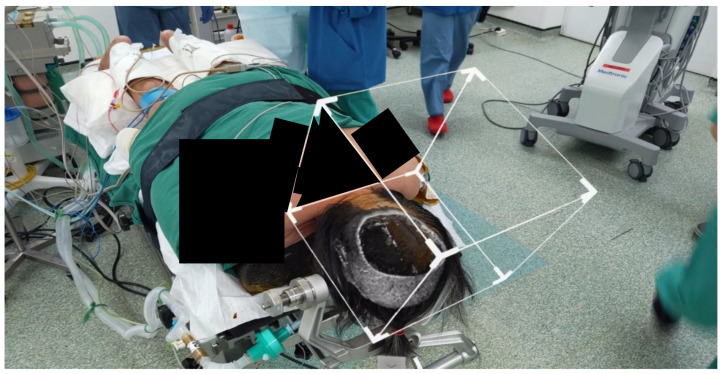
Rendered 3D image superimposed using the pinna of bilateral ears and temporomandibular joints prior to surgical draping.

Intra-operatively, the patient’s head was clamped and placed in prone position to aid visualization. Stealth S8 station was used for registration and neuronavigation. Prone positioning resulted in multiple attempts to register adequately. Eventually, an accuracy was achieved to 2.2 mm. The standard landmarks of external auditory meatus, medial and lateral canthus and nasion were checked to ensure accuracy of the neuronavigation. The rendered 3D image was then superimposed using the pinna of bilateral ears and tip of the nose as the landmarks to be matched to. Figure 2 shows the rendered image superimposed onto the patient with slicing at the level where the tumour was visible. 

Standard draping and preparation procedures were then followed. Unfortunately, after the draping, skin landmarks used for initial mapping were no longer available. A skin flap was raised based on previous op. As the MRI rendering allowed for visualization of the craniotomy, the edges of the original craniotomy were now used for matching of the rendered image. 

**Figure 3:**
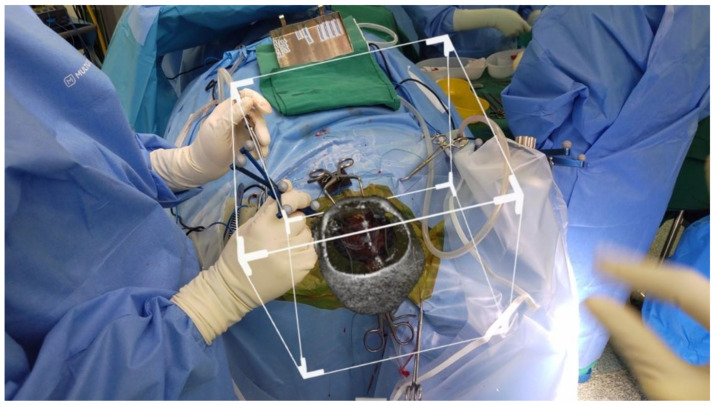
Rendered 3D image superimposed using prior craniotomy after surgical draping. Slicer used to show the tumour. Navigation probe localizes the tumour at the same location as seen through the HoloLens 2.

The bone flap was removed. Dura was reflected medially, and brain was retracted laterally to allow for an approach to the falcine lesion. The neuronavigation probe was then placed at the superficial edge of the flax and introduced to reach the lesion by the surgeon. As shown in Figure 3, the site probe coincided with that of the neuronavigation probe with no drift errors. The surgery was completed uneventfully. 

### 3.2. Case 2

**Figure 4:**
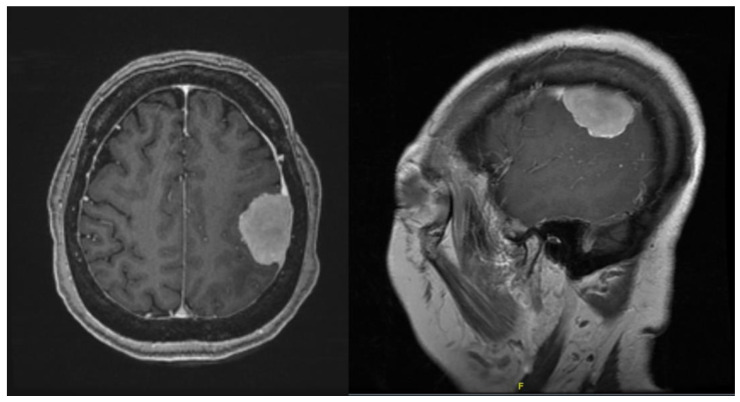
Contrasted T1 MRI showing homogenously enhancing frontal convexity meningioma.

The next patient was a 67-year-old female presenting with right-sided weakness. On examination, she was noted to have grade 3/5 on the right upper and lower limb. MRI brain with contrast showed a 3.6 × 2.7 × 1.4 cm left frontal convexity tumour with features consistent of that being a meningioma (Figure 4). Patient was counselled for craniotomy and resection of tumour under general anaesthesia. 

**Figure 5:**
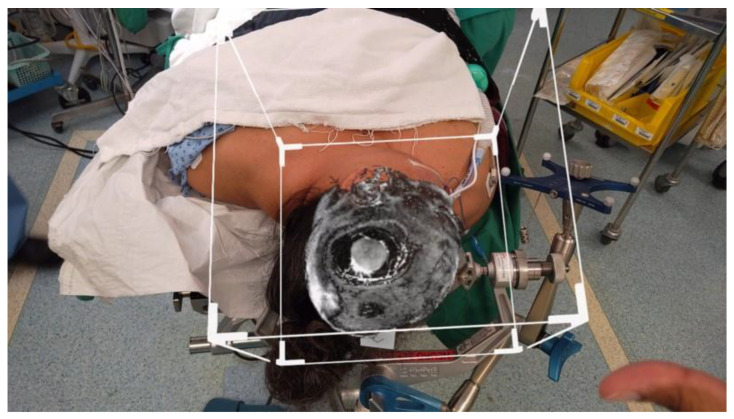
Rendered 3D image superimposed using the pinna of bilateral ears and tip of the nose.

As shown in Figure 5, patient was placed in a supine position with head turned to the left side with the head clamped. Standard neuronavigation was used for registration and surface marking of tumour. Subsequently, we used the HoloLens for 3D image rendering, which was superimposed using the tip of the nose and the pinna of the bilateral ears. As shown in Figure 5, the slicer was able to visualise the anterior and posterior boundaries of the tumour well. This correlated with the boundaries reflected by the neuronavigation. We did not perform a quantitative evaluation of accuracy of the HoloLens versus standard neuronavigation, as the purpose of this series was to evaluate the feasibility of using the HoloLens intraoperatively. 

**Figure 6:**
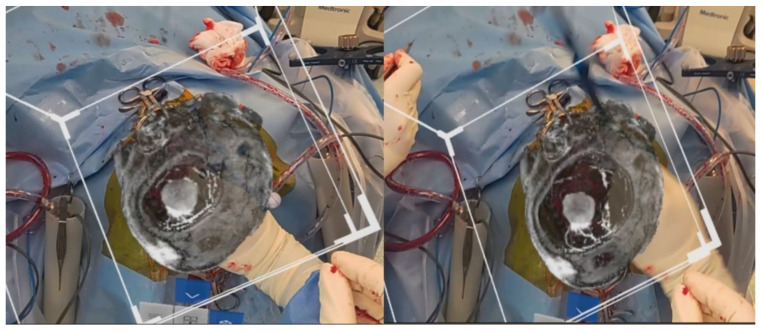
Rendered 3D image superimposed using prior craniotomy after surgical draping. Slicer used to show the tumour.

After draping, there was no movement of the image, and the correlation with the neuronavigation remained. Figure 6 shows the slicer showing the tumour intra-operatively, which correlated with the intra-op findings after the dura was opened. Surgery proceeded uneventfully. Patient recovered well post-operatively.

### 3.3. Case 3

**Figure 7:**
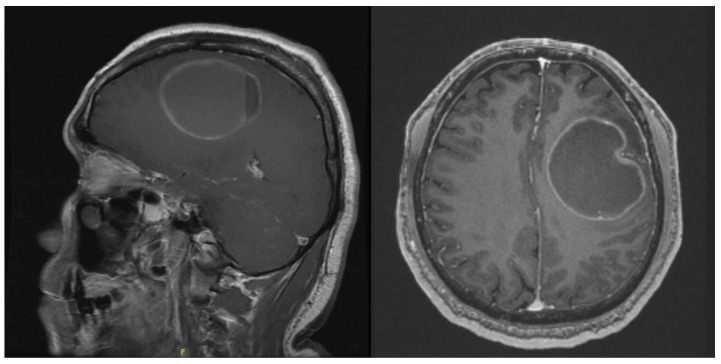
Contrasted T1 MRI showing the intra-axial cystic tumour.

The next patient was a 57-year-old male presenting with right-sided weakness and short-term memory loss. On examination, he was noted to have grade 4/5 on the right upper and lower right limb. MRI brain with contrast showed a 6.6 × 5.2 × 5.1 cm intra-axial tumour in the left fronto-parietal region (Figure 7). Diffusion imaging tractography (DTI) was also performed on this patient. 

**Figure 8:**
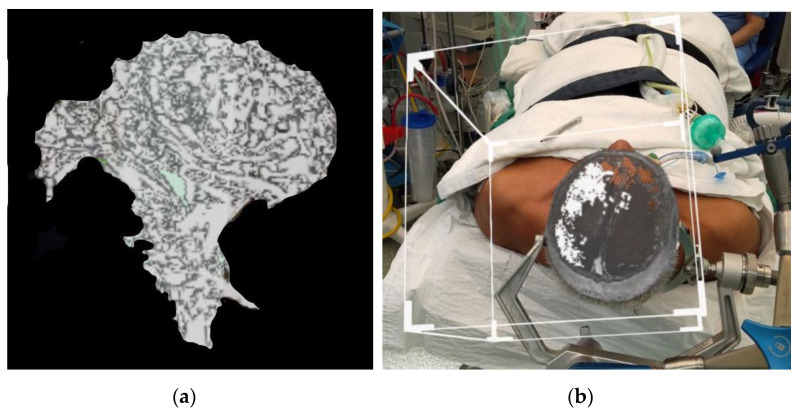
(**a**) Rendered 3D view of the white matter tracts generated. (**b**) Rendered white matter tracts with T1 image.

The images were processed using Omniscient^®^ tractography software. Patient was counselled for craniotomy and resection of tumour under general anaesthesia. These tracts were exported to HoloLens 2. Figure 8a shows the tracts being projected alone; Figure 8b shows the tracts being projected onto the tumour prior to draping. 

**Figure 9:**
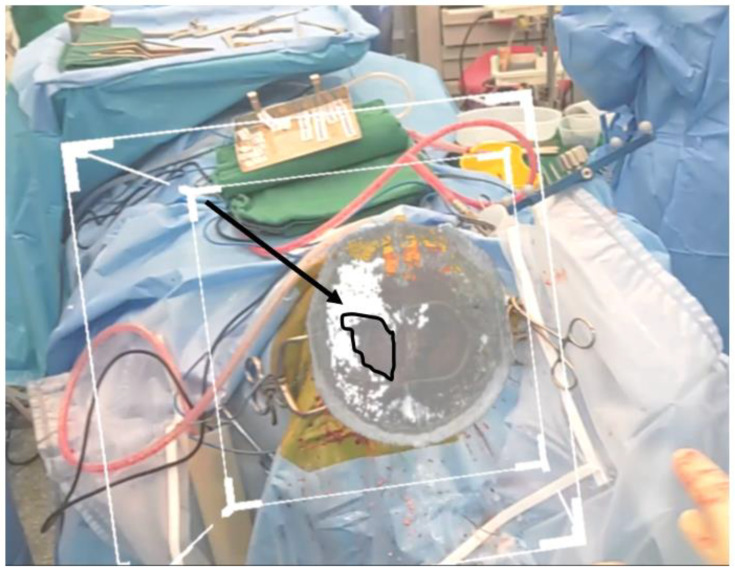
Rendered 3D image superimposed using prior craniotomy after surgical draping. Slicer used to show the tumour and tracts. The black arrow is pointing towards the white matter tracts, and the black outline is used to identify the tumour.

As shown in Figure 9, the patient was placed in a supine position. Standard neuronavigation was used for registration and surface marking of tumour. Subsequently, we used the HoloLens for 3D image rendering, which was superimposed using the tip of the nose and the pinna of the bilateral ears. As shown in Figure 9, the slicer was able to visualise the anterior and posterior boundaries of the tumour along with the superimposed white matter tracts. This correlated with the boundaries reflected by the neuronavigation.

## 4. Discussion

The neurosurgical community has been fortunate. Despite the slow and pendulous interest in adopting new technology, parallel developments in the mixed reality (MR) realms have allowed for the rekindling of interest in adapting new MR gadgets [1,5,19,21,22,23,24,25,26,27,28]. HoloLens 2 has brought MR back to our theatres, providing a significant leap forward and overcoming the disadvantages of the previous VR and AR systems. 

In this short case series, we have assessed the feasibility of using a mixed-reality device HoloLens 2 in neurosurgical operations. All neurosurgical patients that are undergoing surgical resection of intra- or extra-axial tumours usually require pre-operative imaging, which can be used for intra-operative neuronavigation. The first step in this study was to evaluate if any further modifications in the imaging protocols are required to render these images on the MR platform. MRI files in Digital Imaging and Communications in Medicine (DICOM) format could be easily uploaded onto the Microsoft Cloud platform without requiring any modification. The slice size was not a limitation when rendering the images on the HoloLens 2 VSI platform. Using 1 mm slices that are obtained as part of standard neuronavigation, the resolution of rendering was adequate for visualization. For the purposes of the study, rendering was performed using T1 weighted images with contrast for all three patients. 

We attempted to see if rendering of other MRI sequences can be performed. This was accomplished in the third case where processed DICOM format DTI images were exported. The software was able to render good 3D images of the white fibre matter tracts (Figure 8 and Figure 9) which were easily visualized intra-operatively. As we were limited by the processing software used in this case, we were unable to differentiate between the tracts, and we were unable to achieve colour rendering of the white matter tracts. We are planning a subsequent study to evaluate different tractography software on the MR platform. The flexibility of using any DICOM images will make HoloLens 2 an attractive alternative device, as it would not be limited by the requirements of each neuronavigation system. 

HoloLens 2 is a hands-free MR device. It eliminates the need for mental image processing from a 2D display to the 3D anatomy in operating room. It does not require constant checking of the neuronavigation on a distantly displayed screen while operating. While the 3D-rendered images are attractive, their safety and efficacy, inter-user variability and application in the operating theatre remain unassessed. A systemic review by Cho et al. [29] revealed nearly 26 cases of intracranial neurosurgical cases where virtual reality or AR was used. The first-generation HoloLens was shown to have higher registration and planning time when used in 25 patients. This same system was found to be clinically useful in six patients in localizing superficial tumours and optimizing incision planning and craniotomy [30]. However, the system could not be used once the microscope was introduced. 

We faced similar issues in our study. In all three cases, good-resolution-rendered images were visible as long as a microscope was not introduced. The HoloLens 2 had to be removed if the primary operator of the device required the use of the microscope. However, there were several features on the HoloLens 2 which proved handy. The presence of a two-handed, ten-finger fully articulated model and direct manipulation capability allowed for manipulation of the rendered image without loss of sterility during the operation. The see-through holographic lenses allowed the user to change visualization between the real world and the rendered image. This allowed the primary user of the device to be able to operate while wearing the device. Other features such as real-time eye tracking, world-scale positional tracking and real-time environment mesh allowed for little to no drift in the positioned image before and after draping. 

Virtual Surgery Intelligence (VSI) provides the software the capability to image render and to overlay in real time during an operation. VSI is a software designed specifically for clinical use cases and has been undergoing development over the last few years. It can convert any computed tomography (CT) or magnetic resonance imaging (MRI) file in Digital Imaging and Communications in Medicine (DICOM) standard into a 3D rendering which can then be overlayed onto the patient during the operation. It can also display 3D printing files in STL format without additional rendering. Users are granted full control over these images, including positioning, size, contrast, opacity, and image threshold manipulation. A “slicer box” is also available for the user to look inside the 3D rendering. This slicer box was very useful in Case 1 where the tumour was deep seated. The slicer box could be used to “slice” the image in any plane, beyond the limited axial/sagittal/coronal planes in standard neuronavigation. This was helpful even for superficial tumours, especially to visualize the relation of the tumour with the surrounding neurovascular structures. Due to usage of the 1 mm slice MRI, there was no depreciation in image quality when the slicer was used in any desired plane. 

### 4.1. User Training and Pre-Operative Planning

Bernard and Bijlenga Pr [21] have reported that a major challenge for virtual, mixed, and augmented reality technologies is being able to control the user perception of distinguishing virtual models from reality and preserving patients’ safety from errors, information overflow, or inattentional blindness. Development in the field of mixed reality must comply with health care needs regarding safety, precision, and reliability, as well as personalized care, data protection, and costs. HoloLens 2 provides a relatively easy platform to use. No extra training session hours were required for the purpose of the study. 

The user interface for VSI is straight forward and relatively easy to use. A short training session was conducted for the operating team prior to surgery, and the team was allowed to familiarize themselves with the device and user interface. The average time taken for a user to become familiar with the controls was 30 min without prior experience with a mixed-reality device. The results of user friendliness using a questionnaire are currently being analysed using a larger sample size in another study. 

The process of image overlay was relatively straightforward. Users will identify and match reference points on the patient with that on the 3D rendering for both size and directional overlay. Once the image overlay has been positioned correctly, it will stay in place via the stereotactic cameras on the HoloLens 2, allowing the image to be fixed in space regardless of the movement of the user. Spatial anchors are important for this to function consistently and to prevent image drift or swim. It was relatively easy to position the rendered image onto the position. Registration in prone position with a conventional neuronavigation system is often difficult due to landmarks such as nasion, glabella and zygoma facing the floor. Contrarily, the 3D-rendered image completely eliminates the need for pre-defined landmarks. Other alternatives such as bilateral pinna and temporomandibular joints were used in this case to ensure accuracy of the image.

However, manual positioning of the rendered image lead to issues with accuracy. This study did not look at the accuracy of the positioned image with standard neuronavigation. As mentioned in the Materials and Methods section, we did not change the exposure of the operating field to allow for visualization of landmarks, which were used to initially position the image prior to draping. In Case 1, we had to use the margins of craniotomy to confirm the position of the image. This would not be possible in cases which have not been operated on before. Furthermore, for biopsy of deep-seated lesions where accuracy is paramount, it would be not possible to propose a MR platform to use for navigation. 

The position of fixed points such as the head clamp provide good spatial anchors for the HoloLens 2 to identify. As the navigation arm is exposed even after draping, the spatial anchors were not lost, and there was no movement of the image. We acknowledge that in this study, we have not provided any quantitative data on image drift. 

Autoregistration of the 3D-rendered image is currently under development. An upcoming software update for VSI will include the ability for auto-mapping, where the software will automatically map anatomical landmarks on the patient and correlate those with anatomical landmarks on the 3D image, thus negating the need for the user to perform a manual overlay. This will significantly improve image accuracy as well as image stability by using the patient’s own anatomical landmarks as spatial anchors rather than the surrounding objects. 

### 4.2. Correlation with Neuronavigation

Our three cases show good visualization of images intra-operatively, before and after draping of the patients. The question of whether HoloLens 2 can replace the gold standard neuronavigation remains unanswered. There have been few reports that have been published to assess its accuracy. Qi et al. [19] have recently published a study to assess the technical feasibility of using a MR device for neuronavigation. In this study for 37 patients, multimodal imaging–based holograms of lesions, markers, and surrounding eloquent structures were created. A point-based registration was used for the purposes of the study. The overall median deviation was 4.1 mm, and 81.1% of the lesions localized by MR were found to be highly consistent with standard neuronavigation (deviation < 5.0 mm). Koike et al. [1] showed that using mixed-reality projection mapping had excellent accuracy. Most of these studies have used marker-based imaging protocols. 

The purpose of this study was not to describe the differences in neuronavigation using optical navigation and mixed reality. However, subjectively, there was good correlation with the localization of the tumour. As displayed in Figure 2 and Figure 5, neuronavigation correlated well with tumour localization provided by the HoloLens 2. As the surgeon wearing the HoloLens 2 was not required to look away from the surgical field, instruments could be advanced easily to identify the location of the tumour with seamless switching while looking through the lens or direct visualization. 

Our unit is further developing protocols to measure absolute drift in localization between neuronavigation and MR. While this case did not require assessment of tumour boundaries, detailed studies are required to understand limitations in the system in defining tumour margins, depth of tumour, and registration of instruments with MR. It would be prudent to assess the accuracy without involving a marker-based imaging system, as that would involve changing current imaging protocols. Our work has recently been accepted for publication. We have looked at the assessment of accuracy of mixed reality devices for neuronavigation, proposing a generalizable method and results of the current HoloLens 2 platform using VSI as the software. 

### 4.3. Surgeon Fatigue and Visualization

**Figure 10:**
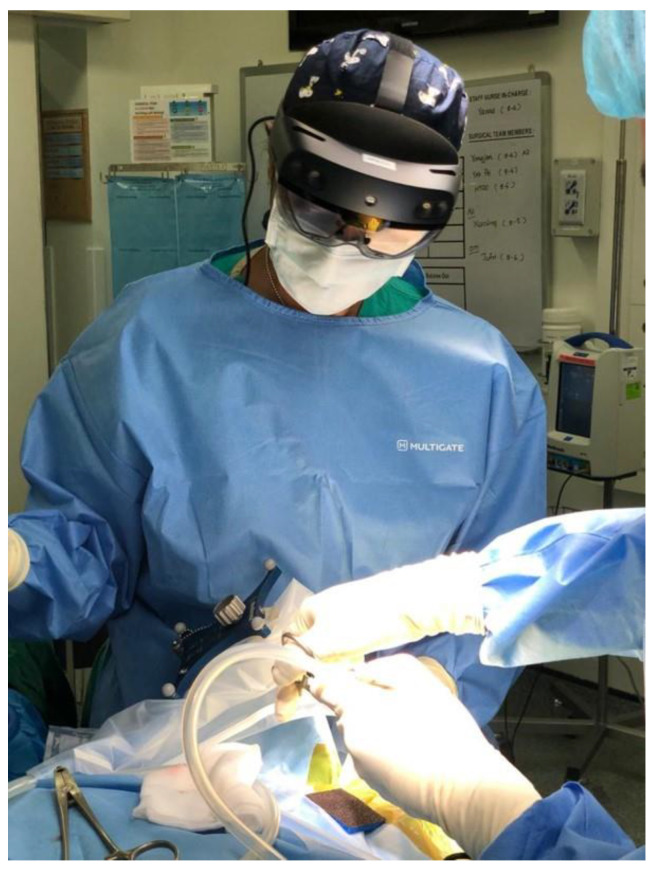
Primary surgeon wearing HoloLens 2 throughout the operation.

The lens was light and, after 2 h of constant wearing (Figure 10), did not cause any fatigue to the operator. Vision could be easily adjusted while looking through the lens or directly at the operative field, allowing for easy manipulation of the surgical field if the HoloLens was not required. 

Direct illumination of the operative fields by the theatre lights decreased the visibility of the rendered image despite increasing the brightness to maximum. This often required the assistant to move the lights away if visualization through the HoloLens 2 was required. 

### 4.4. Review of Images and Videos

The HoloLens 2 allowed for static pictures and videos of the rendered image. The users in this case series highlighted the ability to use these pictures and videos as a teaching tool to better understand the relationship between the tumour and surrounding neurovascular structures. The pictorial figures used in this study were obtained using this function of HoloLens 2. 

### 4.5. Limitations

The authors acknowledge that this is a very small heterogenous series to assess the usability of HoloLens 2. While the authors assessed the feasibility of using this device, the study does not provide any quantitative data on the accuracy of the rendered image and its correlation with neuronavigation. The study did not perform a qualitative evaluation amongst the users to ascertain their review in using the platform.

## 5. Conclusions

Recent advances in mixed reality systems have attempted to challenge the standards of pre-operative planning and registration in neurosurgery operating theatres. We presented a short series of three patients to understand the feasibility of using HoloLens 2 in neurosurgery operations without requiring modifications of the existing imaging protocols. The authors have shown that HoloLens 2 is a user-friendly device that provides an alternative platform for intra-operative visualization of neurosurgical pathology. The ability to visualize any image slice in any image allows the surgeon to understand the relationship of the pathology with the surrounding structures. Further work is still required to quantitatively assess the accuracy of the platform with currently available gold standard neuronavigation systems.

## Data Availability

Not applicable.
